# A Plan-Do-Study-Act Cycle to Enhance Operational Efficiency in a Newly Established *Paediatric* Cardiac Operating Room

**DOI:** 10.1093/icvts/ivag006

**Published:** 2026-01-27

**Authors:** Maryam Ali, Shazia Mohsin, Muneer Amanullah, Fatima Ali, Babar Sultan Hasan

**Affiliations:** Sindh Institute of Urology and Transplantation (SIUT), Division of Cardio-thoracic Sciences, Karachi, Sindh, 74000, Pakistan; Sindh Institute of Urology and Transplantation (SIUT), Division of Cardio-thoracic Sciences, Karachi, Sindh, 74000, Pakistan; Sindh Institute of Urology and Transplantation (SIUT), Division of Cardio-thoracic Sciences, Karachi, Sindh, 74000, Pakistan; Sindh Institute of Urology and Transplantation (SIUT), Division of Cardio-thoracic Sciences, Karachi, Sindh, 74000, Pakistan; Sindh Institute of Urology and Transplantation (SIUT), Division of Cardio-thoracic Sciences, Karachi, Sindh, 74000, Pakistan

**Keywords:** operating room efficiency, first case on-time start, turnover time, case cancellation rate, quality improvement, low- and middle-income countries

## Abstract

**Objectives:**

This quality improvement project aimed to enhance operating room efficiency in a newly established paediatric cardiac operating room by focusing on key performance indicators (KPI), including (1) first case on-time start (FCOTS), (2) turnover time (TOT), and (3) case cancellation rate through targeted interventions.

**Methods:**

This initiative was conducted at the Division of Cardiothoracic Sciences, Sindh Institute of Urology and Transplantation (SIUT), from July 2023 to June 2024, using a stepwise Plan-Do-Study-Act (PDSA) methodology for each KPI, including baseline data, interventions, and outcome analysis. Root Cause Analysis with Fishbone and 5 Whys identified gaps, guiding targeted improvements. Weekly performance was tracked on a spreadsheet, and messaging updates were provided, while control charts and feedback sessions ensured progress. Leadership maintained accountability. Pre- and post-intervention results were compared using control charts for FCOTS, case cancellation rate, and for TOT.

**Results:**

Following implementation of targeted QI interventions, FCOTS compliance improved from 50% to 91%, mean TOT decreased from 34 to 27 minutes, and case cancellation rate dropped from 30% to 7%.

**Conclusions:**

Significant improvements in operating room efficiency can be achieved through practical, scalable quality improvement methods using available resources. Our findings support the implementation of KPI-driven QI models in similar resource-limited paediatric surgical settings.

## Graphical abstract

**Figure:**
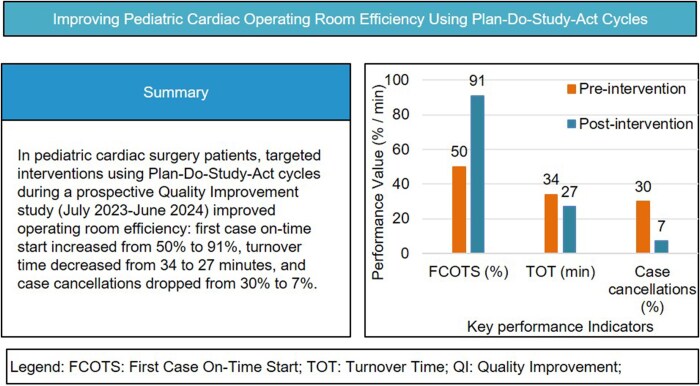

## INTRODUCTION

Operating room (OR) efficiency is crucial in paediatric cardiac surgery, where even minor delays can impact outcomes and strain resources.^1^ In paediatric patients, prolonged fasting, delays, and cancellations increase family stress and health risks, such as dehydration and electrolyte imbalances.^1^ Efficient OR management reduces these risks by minimizing waiting times, optimizing resources, and ensuring timely procedures.^2^

Global literature identifies strategies like structured scheduling, improved coordination, leadership engagement, and technology-assisted workflow optimization as key enablers of timely starts, reduced turnover, and improved performance.^1^^,^^3^^,^^4^ Metrics like first case on-time start (FCOTS) and Lean Six Sigma methodologies have shown success in improving turnover time (TOT).^2^^,^^5^ However, most evidence originates from high-income countries (HICs), where healthcare systems are mature and efficiency is often defined by financial metrics such as cost containment and profitability.^6^^,^^7^

No single tool or metric has been universally validated for evaluating operating-room efficiency across settings. This highlights the need for tailored approaches in resource-limited environments.^8^^,^^9^ In low- and middle-income countries (LMICs), particularly public hospitals, the focus is on resource optimization, equity, and long-term service strengthening, rather than profitability. Our study did not evaluate financial improvement, but future projects will integrate financial performance indicators to assess overall operational efficiency.

In our newly established paediatric cardiac OR, challenges like late starts, cancellations, and prolonged fasting times disrupted workflow and scheduling. Applying QI principles, we addressed system-level barriers in FCOTS, TOT, and case cancellation rate. These challenges underscored the need for a structured, data-driven QI approach to enhance operational performance, teamwork, and process reliability.

## METHODOLOGY AND DATA COLLECTION

### Setting and design

This QI project was carried out in the newly established cardiothoracic services at the Sindh Institute of Urology and Transplantation (SIUT) at the Division of Cardiothoracic Sciences, a public-sector tertiary hospital in Pakistan, from July 2023 to June 2024 using the Plan-Do-Study-Act (PDSA) cycle as its guiding framework that supports stepwise planning, implementation, evaluation, and refinement.^10^ Each cycle involved planning a change, executing it on a small scale, studying its outcomes through data analysis, and acting on the findings to modify, adopt, or sustain the intervention. This systematic approach allowed the multidisciplinary team to evaluate the effectiveness of each intervention phase, address context-specific barriers, and ensure that improvements were data-driven and sustainable.

### Aim statement

The project sought to enhance OR efficiency by targeting 3 key metrics through individual improvement goals with benchmark targets adapted from literature.

*FCOTS*: To increase from 50% to ≥90%^11^ within 6 months.

*TOT*: To reduce from 34 to 25 minutes^7^ within 6 months.

*Case cancellation rate*: To decrease from 30% to <10%^12^ (optimum 5%) within 6 months.

Each KPI was assessed in baseline and pre-intervention results were compared with the post-intervention phases.

### Brief definitions of key performance indicators (KPIs)

- *FCOTS*: Defined as the first elective case wheeled into the operating room by 08:45 h.
- *TOT*: The time between one patient exiting and the next patient entering the same operating room.
- *Case cancellation rate*: Refers to elective cases scheduled but not operated on the same day. Full definitions and formulas of KPIs are provided in the **Supplementary Appendix**.

### Rationale for baseline month selection and KPI choice

Baseline months were chosen according to when each performance metric became reliably measurable in our programme. Early in the paediatric cardiac service, with only 2 elective surgeries per week, problems like cancellations or turnover time were uncommon and inconsistent. As surgical numbers and complexity grew, these issues became measurable, so baselines were staggered (FCOTS: July 2023, cancellations: September 2023, TOT: November 2023). Each baseline was set prior to using the first month with complete, auditable data to avoid bias. We focused on 3 KPIs most relevant to patient flow, resources, and scheduling in our new, evolving paediatric cardiac programme.

### PDSA cycles timings

To ensure valid comparisons and avoid overlap, baseline and intervention phases for each KPI began at different times.

FCOTS:

- Baseline: July 2023
- Intervention: August 2023
- Post-intervention: September 2023 to June 2024

Case cancellation rate:

- Baseline: September 2023
- Intervention: October 2023
- Post-intervention: November 2023 to June 2024

TOT:

- Baseline: November 2023
- Intervention: December 2023
- Post-intervention: January 2024 to June 2024

### Plan (P) and Do (D) phases

**Figure 1.**
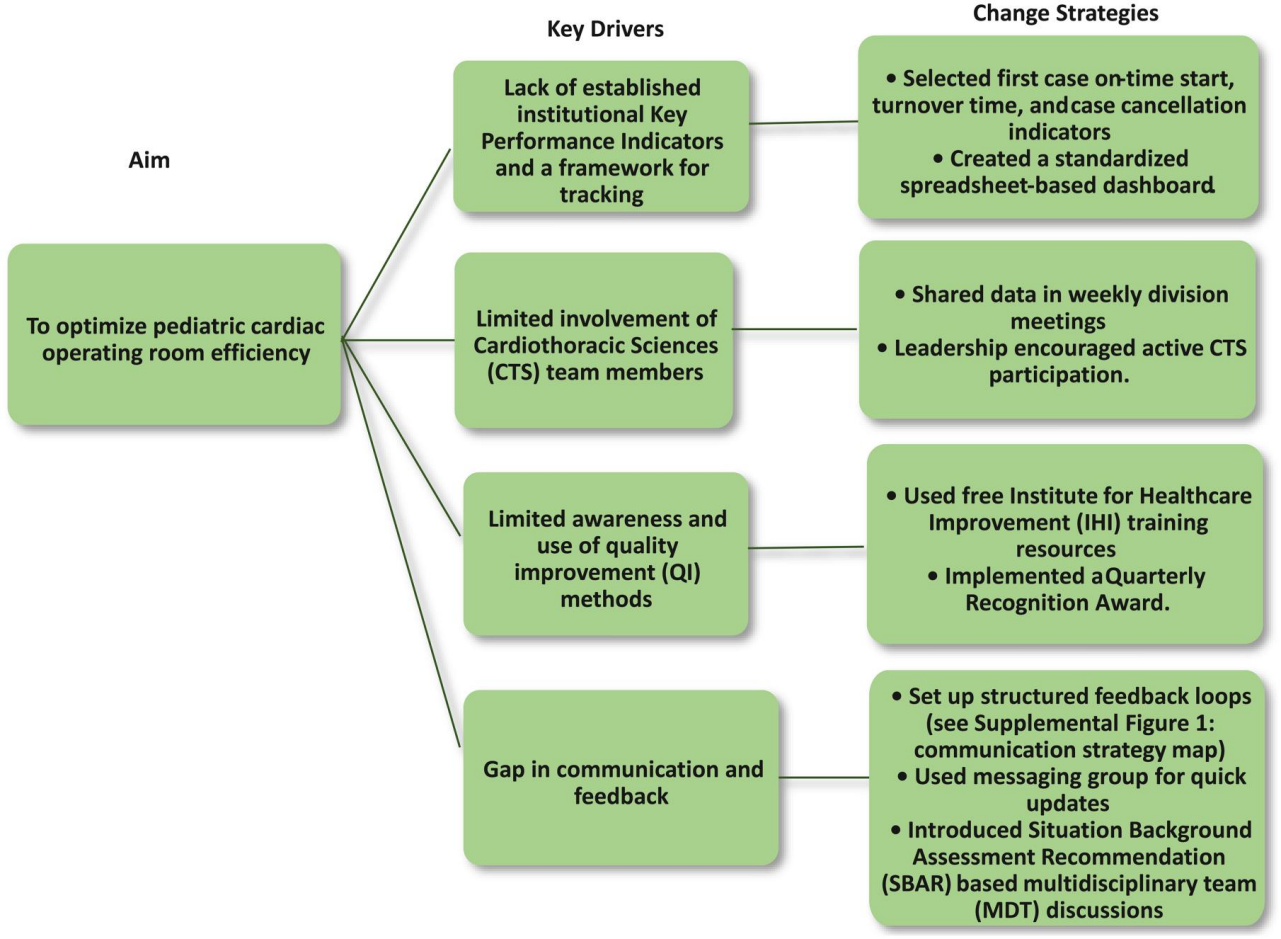
Key Driver Diagram. Displays the primary drivers and change ideas that supported improvement in OR efficiency.

**Figure 2.**
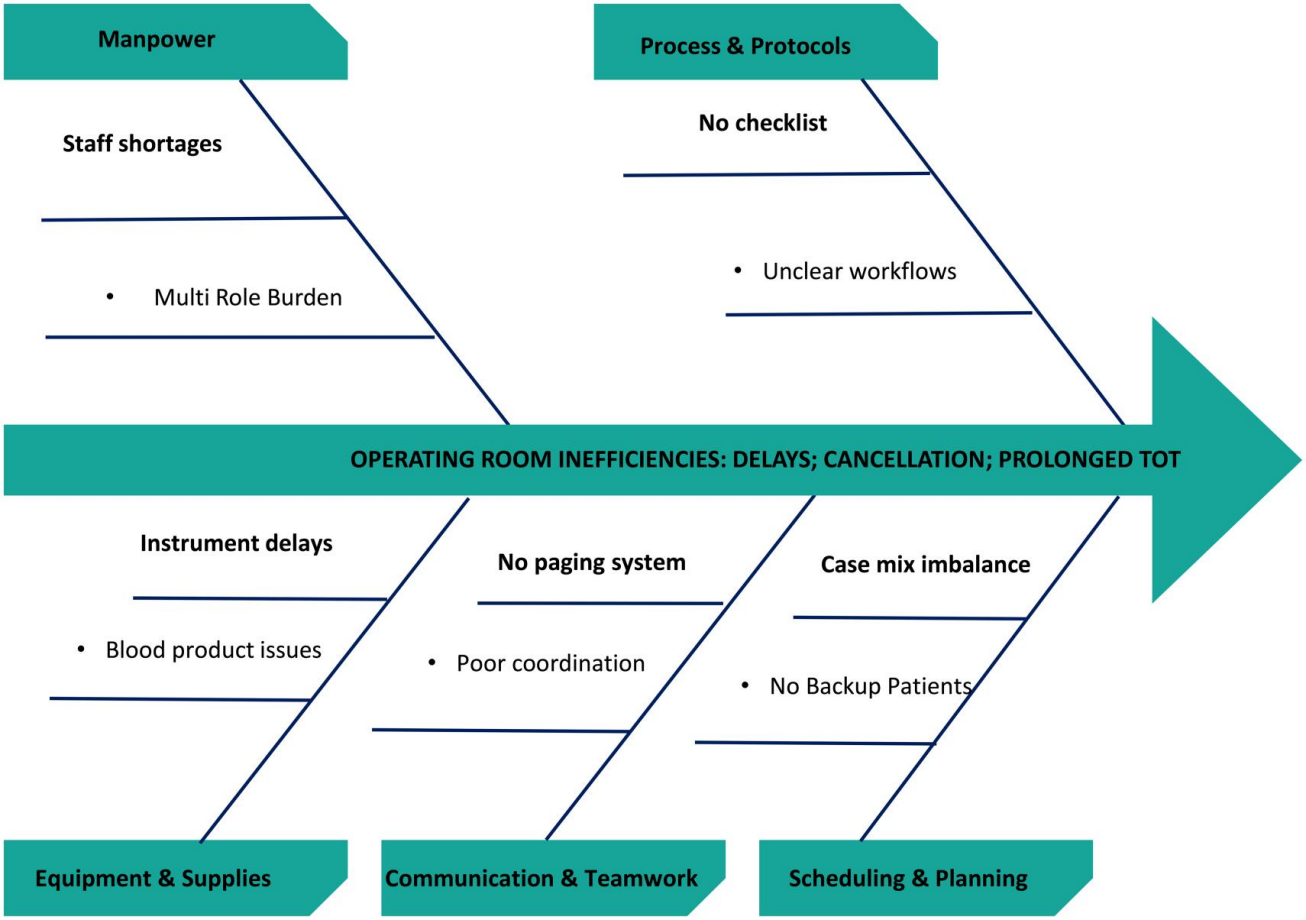
Fishbone Diagram Identifying Root Causes of OR Inefficiencies. Illustrates the main categories of causes contributing to inefficiencies in paediatric cardiac OR workflow.

Baseline data for selected KPIs were collected using standardized spreadsheets, with weekly means for monitoring and monthly averages for trend visualization. Shewhart control charts to analyse process performance for each indicator: an Individuals (I) control chart for continuous turnover time data and p-type attribute charts for case cancellation rate and first case on-time start. Control limits were computed using standard SPC formulas, with UCL and LCL defined as ±3 standard deviations from the process mean (I-chart) or using binomial variance estimates (p-charts). Key strategies are summarized in **Figure 1** and **Table 1**. Root cause analysis using fishbone diagrams and the 5 whys (**Figure 2** and **Figure S2**) identified key issues. A patient flow chart (**Figure S3**) mapped the journey from clinic to ICU. Targeted interventions for each KPI were implemented between August and December to enhance teamwork and sustain continuous improvement. Key strategies are summarized in **Figure 1** and **Table 1**.

1. **First case on-time start (FCOTS):** 
**Contributing factors and targeted interventions:** 
  - **Timely patient arrival and complete NPO status:** 
    Frequent delays due to late patient arrival and incomplete fasting were addressed by mandating admission the afternoon before surgery to complete preoperative workups and confirm results. The paediatric cardiac surgery registrar communicated NPO status to the on-call medical registrar, and the final OR list was shared a day earlier to streamline preparation.
  - **Ensuring pre-procedural availability of blood products from the blood bank:** 
    Arranging blood products and donors for whole blood was a major challenge, especially for out-of-town patients. Our institution follows a direct replacement system, where designated donors, usually the donor’s family or friends, must donate before elective surgeries. Delays occurred when donor arrangements were incomplete. To streamline the process, families were counselled about blood requirements during scheduling, donors were contacted in advance, and coordination with the blood bank ensured early morning staff availability. The on-call registrar arranged porters to collect blood products each morning, improving efficiency.
2. **Turnover time (TOT):** 
**Contributing factors and targeted interventions:** 
  - **Human resources:** 
    OR staff shortages delayed case preparation as the limited team managed multiple tasks, including patient transfer, room cleaning, instrument delivery, OR setup, and preparing the next patient. This was challenging in the newly established cardiac OR, where surgeries are more resource- and time-intensive. Initially, securing additional staff was difficult, but rising caseloads gained leadership support. Workflow was optimized by redistributing tasks: porters handled blood products and instruments, anaesthesia assistants checked equipment, and nurses led OR table setup with parallel patient verification. These changes, along with added staff, reduced preparation time and improved satisfaction.
  - **Cleaner’s role in minimizing delays in operating room cleaning:** 
    With only one cleaner per shift handling the entire OR and no paging system or personal mobile, locating them for timely cleaning was challenging. We addressed this by involving cleaning staff as part of the team, sharing the OR schedule, and expected procedure completion times. Encouraging a proactive approach rather than a punitive one improved communication and teamwork, significantly reducing OR cleaning delays.
  - **Ensuring prerequisites for the second patient are checked concurrently with OR preparation:** 
    Before the initiative, staff traditionally waited until the OR was ready before checking prerequisites and bringing the next patient to the table, which caused delays. To streamline this process, we adopted parallel workflows: one designated OR staff member began preparing the OR while, at the same time, another staff member who transported the first patient to the ICU, checked the prerequisites for the second patient, and brought them to the OR without delay.
3. **Case cancellation rate:** 
**Contributing factors and targeted interventions:** 
  - **Most causes were avoidable and patient-related, with the most significant being upper respiratory tract infections (URTIs), abnormal blood test results, and non-adherence to preoperative fasting guidelines:** 
    A weekly multidisciplinary team (MDT) meeting reviews upcoming surgical cases in a conference room with Zoom access and a display monitor. Attendance is mandatory for all stakeholders. The surgical registrar and cardiology fellow present cases using a standardized PowerPoint, with echocardiograms shown via Viewpoint by an imaging cardiologist. Relevant investigations are shared, and the primary cardiologist highlights key findings. Senior faculty lead discussions with input from all participants. Final plans are surgeon-led and documented on an MDT decision form signed by cardiology, ICU, and anaesthesia teams. To address rising cancellations, backup local patients are reviewed for potential advancement, patients are informed of their waiting status, and respiratory viral panels, especially during RSV season, are included in preoperative screening.
    Most preoperative screenings were conducted as planned; however, consistent completion on every surgical day could not be guaranteed due to workflow constraints. These constraints primarily involved late surgical admissions, delays in donor blood availability (as families arranged replacement donors), overlapping responsibilities among limited operating room staff for case setup and patient transfer, and occasional CICU bed unavailability. Same-day list changes in a high-volume setting further compressed preoperative preparation time and contributed to variability in screening completion.
  - **Unavailability of CICU beds due to full occupancy by critically ill patients, leading to case cancellations rates.** 
    During multidisciplinary team (MDT) meetings, cardiac cases were initially listed without balancing the case mix, sometimes scheduling 2-3 high-risk surgeries on the same day, leading to prolonged CICU stays and disrupted flow. A consensus-based selection process was introduced using the Risk Adjustment for Congenital Heart Surgery (RACHS-1) methodology^13^ [see **Table S1** (RACHS-1) methodology] and clinical factors such as cyanosis and malnutrition. Administrative staff documented data in standardized spreadsheets, and weekly risk profiles were reviewed and adjusted by team consensus. Finalized schedules were shared with frontline teams, while sonographers documented imaging details (TEE/TTE) to support care continuity.

**Table 1.** Intervention Areas and Associated Change Strategies

S. no.	General key drivers	Change strategies
1.	Absence of institutional key performance indicators (KPIs) and standardized tracking protocol	As there were no institutional KPIs for tracking OR efficiency, we selected internationally recognized standards from the literature and identified benchmarks that aligned with our setting. We implemented a spreadsheet-based tracking system as a central repository for real-time monitoring and analysis of KPIs. Each KPI was clearly defined and organized within the spreadsheet, with columns for data entry, dates, and benchmark comparisons. The sheet ensured consistent data tracking, easy visualization of trends, and immediate identification of areas requiring improvement. It was made accessible to relevant team members to facilitate oversight, collaboration, and accountability.
2.	Insufficient involvement of various members of the cardiothoracic sciences (CTS) division.	To address the insufficient involvement of CTS division members, including perfusionists, OR technologist, and nursing staff, leadership implemented regular data presentations and created a platform for transparent, constructive discussions. This enabled leaders to identify trends, celebrate achievements, and offer actionable suggestions. Additionally, the divisional chair and chief cardiac surgeon took personal accountability, maintained consistent communication, and provided progress updates to institutional leadership and the central QI team to ensure ongoing support and sustainability.
3.	Lack of systematic use of quality improvement (QI) sciences.	To address this, we implemented a multi-faceted strategy to build awareness, skills, and accountability. We used the free Institute for healthcare improvement (IHI) online QI courses for foundational understanding. A QI team was established with responsible, accountable, consulted, and informed (RACI) assigned responsibilities. We organized sessions to share goals and educate the team on the initiative’s importance. Unit-level team leaders were appointed to drive accountability and promote a shared vision for improvement, embedding QI culture across the organization.
4.	Ineffective communication and feedback mechanisms.	This was the biggest challenge in our project. Initially, the gap was addressed by the primary lead, the chief OR technologist, who showed exceptional commitment to the intervention by communicating effectively and setting an example to avoid deviations in care plans and strategy implementation. By leading from the front, it demonstrated the importance of alignment and consistency in care delivery. Over time, these efforts inspired team members to recognize the value of feedback and actively cooperate, allowed us to design and structure a system to effectively close the feedback loop.

Summary of targeted QI changes mapped to system issues and outcomes.

### Study

Post-intervention data collected during August 2023 to June 2024 were analysed to track the impact of the interventions. The outcomes were compared with pre-intervention data to evaluate the effectiveness of the change strategies. The primary outcome was monitored based on the identified KPIs.

Improvement was assessed by using control charts for pre- and post-intervention comparisons.

### Act

After reviewing the outcomes of the implemented changes, a decision was made to maintain the use of key performance indicators (KPIs) to support long-term progress. The interventions demonstrated positive impact and were formally adopted into routine operations. The Spreadsheet-based dashboard created during the initiative remained in use, with the team leader overseeing weekly tracking of metrics.

To improve real-time communication and accountability, a dedicated messaging group was launched, enabling the team to flag performance issues quickly and respond to workflow deviations (**Figure S1**).

Looking ahead, the division chair decided to develop a digital solution featuring an app-based tracking tool and electronic dashboards displayed in both the operating room and intensive care unit, ensuring continuous visibility of performance data.

In parallel, a structured weekly review system was introduced to strengthen data evaluation, encourage collaborative problem-solving, and ensure ongoing leadership involvement in driving operational excellence.

## RESULTS

Following implementation of targeted quality improvement (QI) interventions, 127 first case on-time start (FCOTS), 42 turnover time (TOT) transitions, and 181 elective cases were analysed over 48 weeks.

### First case on-time start (FCOTS)

**Figure 3.**
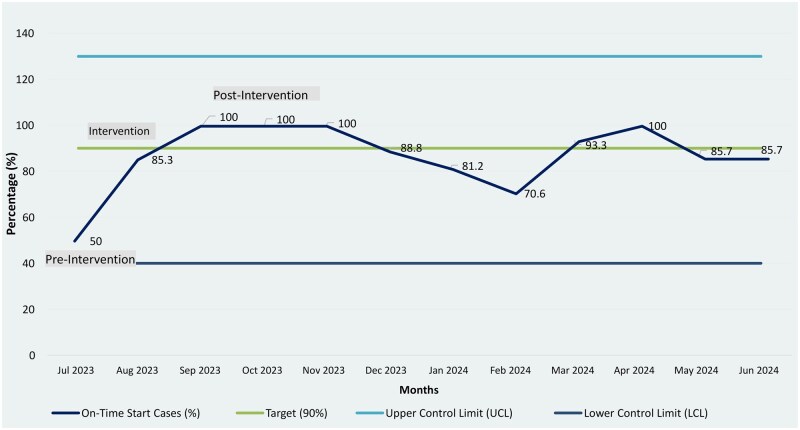
First Case On-Time Start (FCOTS) Control Chart. Control chart showing monthly FCOTS performance pre- and post-intervention.

Compliance improved from 50% (3/6 cases) at baseline (July 2023) to 91% (101/115 cases) post-intervention (September 2023-June 2024) (**Figure 3**).

### Case cancellation rate

**Figure 4.**
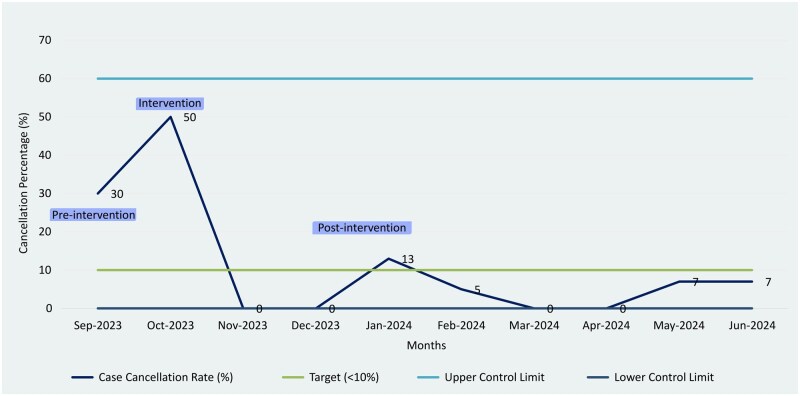
Case Cancellation Rate Control Chart. Displays declining trend in monthly Case Cancellation Rate over the study period.

Rates declined from 30% (3/10 cases) at baseline (September 2023) to 7% (8/161 cases) post-intervention (November 2023-June 2024) (**Figure 4**).

### Turnover time (TOT)

**Figure 5.**
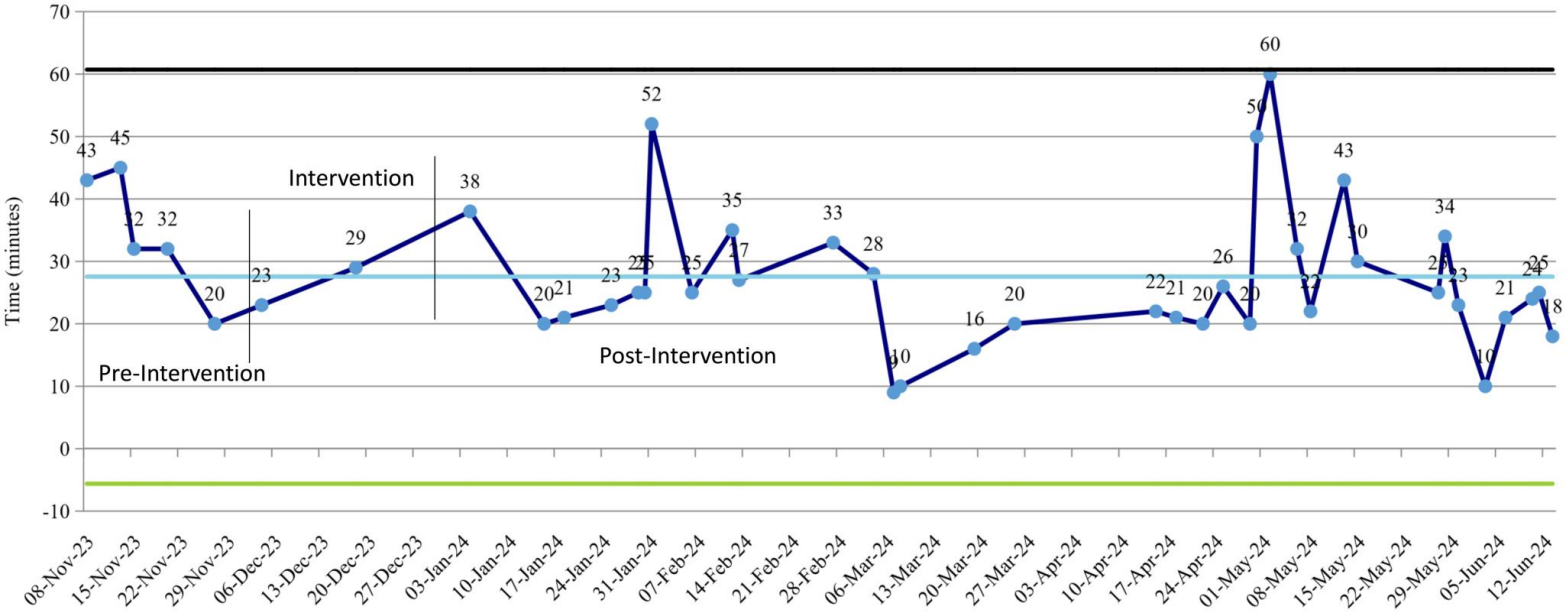
Turnover Time (TOT) Control Chart. Control chart highlighting reduction in mean TOT following QI interventions.

Across the study period, 42 turnover transitions were recorded; 5 occurred during the pre-intervention baseline month, 2 during the PDSA testing roll-out phase, and 35 within the stable post-intervention period. Mean TOT decreased from 34.4 ± 9.4 minutes (*n* = 5) to 26.7 ± 11.2 minutes (*n* = 35) post-intervention (January-June 2024) (**Figure 5**).

## DISCUSSION

This QI initiative is among the first at our institution to track OR efficiency metrics in a newly developed paediatric cardiac surgery unit. Using the PDSA cycle, we achieved improvements in FCOTS, TOT, and case cancellation rate, demonstrating clear benefits for OR performance and patient flow. Limited literature from LMICs on paediatric cardiac OR efficiency makes this work particularly relevant. A learning curve likely contributed to some progress, but the structured PDSA approach ensured that improvements were data-driven and sustainable.

Root cause analysis identified the lack of structured improvement processes and limited staff awareness as key inefficiency factors. These improvements reflect gains in team readiness, coordination, scheduling, and workflow efficiency.

Globally, OR efficiency remains debated. Though various tools have been developed, none is universally effective across all settings.^1^ For example, METERO (Metric for Evaluating Task Execution in the Operating Room) was developed to assess OR task completion and team efficiency.^14^ While METERO is comprehensive and valid in patient safety and efficiency, it is resource-intensive and difficult to replicate. Similarly, Rothstein and Raval^8^ reference metrics from the Canadian Paediatric Wait Times Project,^15^ including off-hours surgery rates, cancellation rates, first case on-time start accuracy, OR utilization, unplanned closures, case duration accuracy, turnover time, and staffing costs. Recent studies highlight machine learning (ML) models in optimizing OR efficiency.^3^

In our context, a newly established paediatric cardiac OR in a public-sector hospital with high patient volume, limited resources, and a shortage of trained staff adopting QI principles was more feasible than using standardized tools alone. We emphasized that “Quality is not an isolated action but a consistent habit,” focusing on low-cost, context-specific solutions to efficiency, process mapping, and fine-tuning.

Similar findings are seen in ICVTS reports, emphasizing the value of structured KPIs and context-specific QI frameworks to strengthen cardiac surgical services. These studies reinforce the importance of evidence-based efficiency strategies such as those implemented in our paediatric cardiac OR.^16^^,^^17^

Recognizing that KPIs alone were insufficient, we promoted continuous improvement and teamwork. Gaps in quality tracking and staff knowledge, attitudes, and practices were addressed. The divisional chair and chief cardiac surgeon led the initiative, appointing the chief OR technologist as team lead. Empowering frontline staff through trust, education, and support, leadership reinforced KPI importance and built skills in tracking, review, and presentation, generating motivation.

To maintain motivation, we implemented a multi-faceted strategy: transparent real-time data via dashboards, weekly team huddles, and a “WOW Bucks” recognition program for high performers. Positive reinforcement, peer acknowledgment, and leadership engagement sustained enthusiasm, ownership, and accountability over 48 weeks.

Teamwork and leadership shifted from fault-finding to shared responsibility. Stepwise KPI rollout prevented overwhelm and supported capacity building. Positive discussions, QI training, and IHI resources fostered accountability without blame.

Although OR efficiency is often linked with costly measures, our low-cost strategies pre-admission planning, standby cases, blood bank coordination, timely cleaning, and staff incentives, improved morale and performance. Stratifying high-risk surgeries optimized CICU bed use.

In LMICs, such data-driven, low-cost approaches supported by digital tools can improve outcomes and sustainability. Importantly, no patient safety indicators, including perioperative mortality, major adverse events, or surgical site infections (SSIs), were adversely affected, as verified through institutional morbidity/mortality and infection control systems. While reassuring, future QI phases will include structured safety metrics as primary outcomes to evaluate both efficiency and safety. Overall, these findings demonstrate that a low-cost, context-specific QI strategy can strengthen paediatric cardiac OR efficiency through structured data tracking and continuous feedback.

### Challenges and limitations

Several limitations were identified in this project. As a QI-based initiative conducted in a newly established operating room, no formal sample size calculation was performed, and improvements were evaluated using control charts rather than inferential statistical tests. The project was limited to the paediatric cardiac surgery unit, with no hospital-wide interventions, which strengthens internal validity but limits external generalizability. Additionally, using a single baseline month for each KPI may have restricted cross-metric comparisons.

Although institutional morbidity/mortality and infection control data confirmed no increase in adverse outcomes, these were not analysed as formal study variables. Unmeasured factors such as workflow refinements or team learning effects may have influenced outcomes. However, consistent leadership and standardized data collection reduce the likelihood that these factors alone explain the improvements. Additionally, a potential Hawthorne effect may have contributed to improved performance, as staff awareness of being observed can transiently influence behaviour.

Future cycles will include 3-6 months of baseline data for greater robustness. The financial impact and psychological aspects of change were not assessed, and long-term sustainability will be reported in a planned follow-up study.

## CONCLUSION

Significant improvements can be achieved even with a practical, low-resource, key driver approach to quality initiatives by implementing targeted interventions and consistent monitoring with dedication.

## Supplementary Material

ivag006_Supplementary_Data

## Data Availability

All data generated or analysed during this quality improvement project are included in this article and its supplementary material. Further details may be made available by the corresponding author upon reasonable request.
