# Supplementary material for: A Plan-Do-Study-Act Cycle to Enhance Operational Efficiency in a Newly Established Paediatric Cardiac Operating Room
Source: Interdiscip Cardiovasc Thorac Surg. 2026 Jan 27;41(1):ivag006. doi: 10.1093/icvts/ivag006 (PMC12864523; doi:10.1093/icvts/ivag006)
Supplement: ivag006_Supplementary_Data [file ivag006_supplementary_data.zip › Supplementary Figure 2.pdf]

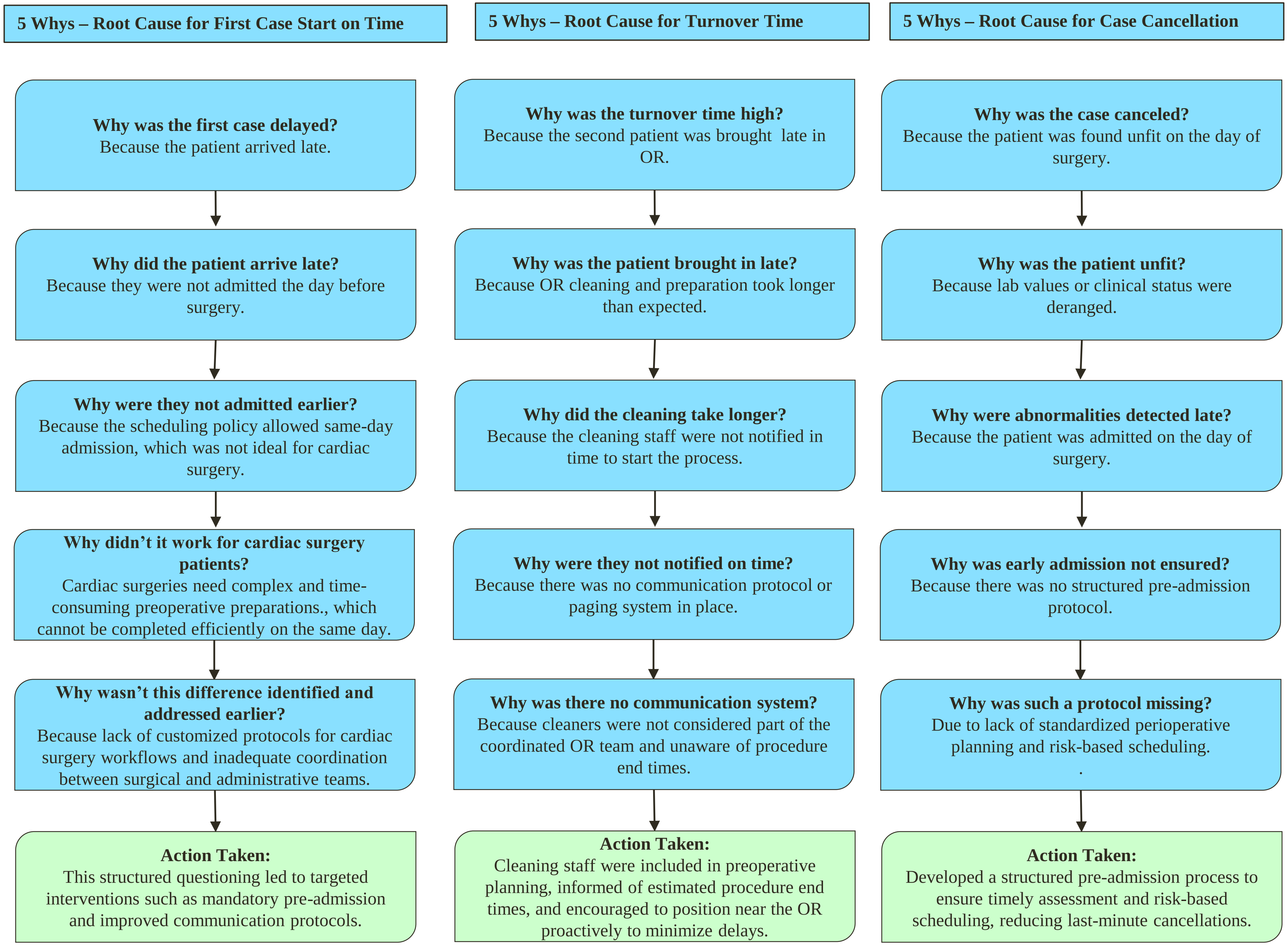
5 Whys – Root Cause for First Case Start on Time
Why was the first case delayed?
Because the patient arrived late.
Why did the patient arrive late?
Because they were not admitted the day before surgery.
Why were they not admitted earlier?
Because the scheduling policy allowed same-day admission, which was not ideal for cardiac surgery.
Why didn’t it work for cardiac surgery patients?
Cardiac surgeries need complex and time-consuming preoperative preparations., which cannot be completed efficiently on the same day.
Why wasn’t this difference identified and addressed earlier?
Because lack of customized protocols for cardiac surgery workflows and inadequate coordination between surgical and administrative teams.
Action Taken:
This structured questioning led to targeted interventions such as mandatory pre-admission and improved communication protocols.
5 Whys – Root Cause for Turnover Time
Why was the turnover time high?
Because the second patient was brought late in OR.
Why was the patient brought in late?
Because OR cleaning and preparation took longer than expected.
Why did the cleaning take longer?
Because the cleaning staff were not notified in time to start the process.
Why were they not notified on time?
Because there was no communication protocol or paging system in place.
Why was there no communication system?
Because cleaners were not considered part of the coordinated OR team and unaware of procedure end times.
Action Taken:
Cleaning staff were included in preoperative planning, informed of estimated procedure end times, and encouraged to position near the OR proactively to minimize delays.
5 Whys – Root Cause for Case Cancellation
Why was the case canceled?
Because the patient was found unfit on the day of surgery.
Why was the patient unfit?
Because lab values or clinical status were deranged.
Why were abnormalities detected late?
Because the patient was admitted on the day of surgery.
Why was early admission not ensured?
Because there was no structured pre-admission protocol.
Why was such a protocol missing?
Due to lack of standardized perioperative planning and risk-based scheduling.
.
Action Taken:
Developed a structured pre-admission process to ensure timely assessment and risk-based scheduling, reducing last-minute cancellations.
