# Supplementary material for: A Plan-Do-Study-Act Cycle to Enhance Operational Efficiency in a Newly Established Paediatric Cardiac Operating Room
Source: Interdiscip Cardiovasc Thorac Surg. 2026 Jan 27;41(1):ivag006. doi: 10.1093/icvts/ivag006 (PMC12864523; doi:10.1093/icvts/ivag006)
Supplement: ivag006_Supplementary_Data [file ivag006_supplementary_data.zip › Supplementary Appendix.docx]

**Supplementary Appendix 01:**

**Key Performance Indicator Definitions:**

1. **First Case On-Time Start (FCOTS):**

FCOTS is define as the time when the patient is wheel into the operating room for the first elective case of the day.

Note: For this project, we decided to start our elective OR Cases at 8:30 AM. A 15-minute grace period is allowed, thus after 8:45 AM the case will be considered late.

1. **Turnover Time (TOT):**

It is defined as the time between surgical cases in the same Operating Room (OR). (Previous Patient-Out to Next Patient-In).

Benchmark: TOT of 25 minutes is categorized as high performance between 26 and 40 minutes-medium performance and above 40 minutes is considered Unsatisfactory.

1. **Case Cancellation Rate:**

Elective surgical case cancellation refers to any surgical case that is booked into the OR list on the day prior to surgery but is not operated upon as scheduled.

**Key Performance Indicator Formulas Used:**

- **First Case On-Time Start (FCOTS) (%)**

**FCOTS** = Number of on time start​ X 100

Total No. of first Cases

- **Turnover Time (TOT) (in minutes)**

**TOT** = First patient exit time – Second patient in time (difference in minutes)

- **Case Cancellation Rate (%)**

**Case Cancellation Rate** = Number of Cancelled Cases​ X 100

Total No. of Scheduled Case

## **Descriptive Statistical Summary**

### ****Table 1. First Case On-Time Start (FCOTS)****

This table summarizes baseline and post-intervention data for First Case On-Time Start (FCOTS) performance, comparing the number of on time versus delayed starts before and after QI implementation.

| **Period** | **On-Time Cases** | **Delayed Cases** | **Total Cases** | **Compliance (%)** |
| --- | --- | --- | --- | --- |
| Pre-intervention (July 2023) | 3 | 3 | 6 | 50% |
| Post-intervention (Sep 2023 - Jun 2024) | 101 | 14 | 115 | 91% |

**Table 2. Case Cancellation Rate**

This table displays the change in elective case cancellations between baseline and post-intervention periods.

| **Period** | **Cancelled Cases** | **Completed Cases** | **Total Cases** | **Cancellation Rate (%)** |
| --- | --- | --- | --- | --- |
| Pre-intervention (September 2023) | 3 | 7 | 10 | 30% |
| Post-intervention (Nov 2023 - Jun 2024) | 8 | 153 | 161 | 7% |

### ****Table 3. Turnover Time (TOT)****

This table presents descriptive data for mean turnover time (TOT) before and after QI interventions.

| **Group** | **Mean TOT (minutes)** | **Standard Deviation** | **Sample Size (n)** |
| --- | --- | --- | --- |
| Pre-intervention (Nov 2023) | 34.4 | 9.4 | 5 |
| Post-intervention (Jan - Jun 2024) | 26.7 | 11.2 | 35 |
