# Supplementary material for: A Plan-Do-Study-Act Cycle to Enhance Operational Efficiency in a Newly Established Paediatric Cardiac Operating Room
Source: Interdiscip Cardiovasc Thorac Surg. 2026 Jan 27;41(1):ivag006. doi: 10.1093/icvts/ivag006 (PMC12864523; doi:10.1093/icvts/ivag006)
Supplement: ivag006_Supplementary_Data [file ivag006_supplementary_data.zip › Supplementary Material Legends.docx]

# Supplementary Material Legends

**Supplementary Figure 1:** **Communication and Feedback Loop for Quality Improvement**
Description of the structured communication framework used for OR coordination and feedback.

**Supplementary Figure 2:** **Root Cause Analysis using the 5 Whys**
Detailed 5 Whys analysis identifying issues leading to OR start delays, turnover inefficiencies, and cancellations.

**Supplementary Figure 3. Process Flow Chart**
*Visual representation of patient flow from the outpatient clinic to the pediatric cardiac operating room and then to the cardiac intensive care unit.*

**Supplementary Appendix :**
Key performance definitions, formulas used, and control charts of FCOTS, TOT, and case cancellations.

**Supplementary Table 1. (RACHS 1) Methodology**

*Methodology for the Risk Adjustment in Congenital Heart Surgery (RACHS-1) classification system.*
