# Supplementary material for: A Plan-Do-Study-Act Cycle to Enhance Operational Efficiency in a Newly Established Paediatric Cardiac Operating Room
Source: Interdiscip Cardiovasc Thorac Surg. 2026 Jan 27;41(1):ivag006. doi: 10.1093/icvts/ivag006 (PMC12864523; doi:10.1093/icvts/ivag006)
Supplement: ivag006_Supplementary_Data [file ivag006_supplementary_data.zip › Supplementary Table 1.docx]

**Supplementary Table 2. Standardized Form for Risk Stratification Using the Risk Adjustment for Congenital Heart Surgery (RACHS-1) Methodology**

| Operation Type | Specification | RACHS Score |
| --- | --- | --- |
| Atrial septum defect (ASD) repair | Patients with an ASD type II, sinus venosus ASD, and partial anomalous pulmonary venous return | 1 |
| Ventricular septum defect (VSD) repair | VSD as dominant diagnosis, potentially with an ASD or patent ductus arteriosus | 2 |
| Tetralogy of Fallot repair | Only primary correction, facultative after a shunt operation | 2 |
| Complete atrioventricular canal (CAVC) repair | Isolated only, with full correction | 3 |
| Arterial switch operation | Patients with an intact septum and with VSD | 3 or 4 |
| Truncus arteriosus (TA) repair |  | 4 |
| Norwood operation |  | 6 |
| Bidirectional cavopulmonary anastomosis (BCPA) |  | 2 |
| Total cavopulmonary connection (TCPC) |  | 3 |

**Abbreviation:** RACHS, Risk Adjustment for Congenital Heart Surgery.

*Adapted from Jenkins KJ, Gauvreau K, Newburger JW, Spray TL, Moller JH, Iezzoni LI. Consensus-based method for risk adjustment for surgery for congenital heart disease. J Thorac Cardiovasc Surg. 2002;123(1):110-118.*
