# Supplementary figures and images for: A Plan-Do-Study-Act Cycle to Enhance Operational Efficiency in a Newly Established Paediatric Cardiac Operating Room
Source: Interdiscip Cardiovasc Thorac Surg. 2026 Jan 27;41(1):ivag006. doi: 10.1093/icvts/ivag006 (PMC12864523; doi:10.1093/icvts/ivag006)

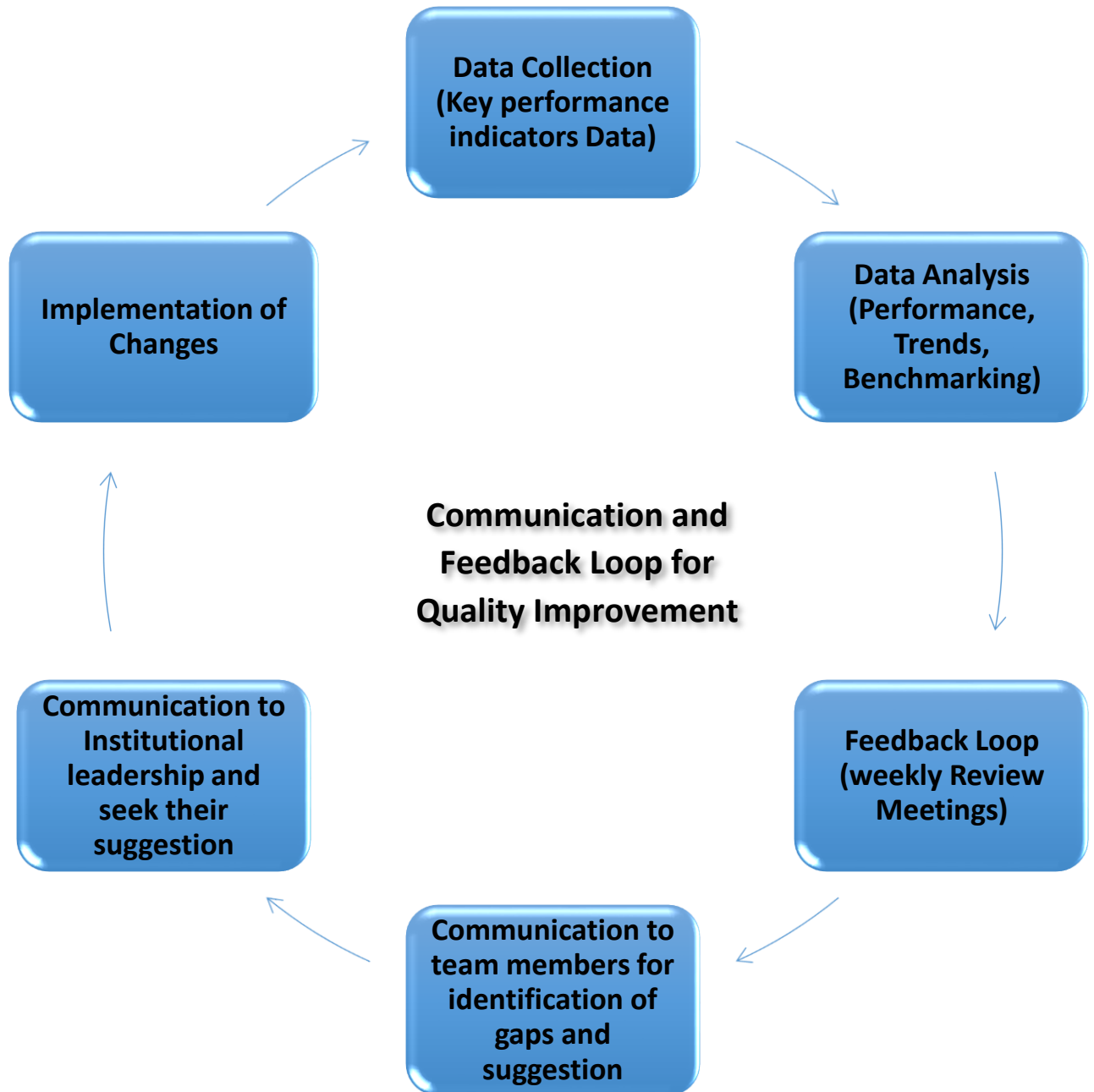

Supplement: ivag006_Supplementary_Data [file ivag006_supplementary_data.zip › Supplementary Figure 1.pdf]
